# Risk factors predisposing to post-traumatic hydrocephalus

**DOI:** 10.3389/fneur.2026.1827667

**Published:** 2026-07-01

**Authors:** Helen Tarousa, Anastasia Tasiou, Maria Karagianni, Iordanis Georgiadis, Kostas N. Fountas

**Affiliations:** 1 Department of Neurosurgery, University Hospital of Larissa, Larissa, Greece; 2 Faculty of Medicine, School of Health Sciences, University of Thessaly, Larissa, Greece

**Keywords:** decompressive craniectomy, hygroma, intraventricular hemorrhage, outcome, post-traumatic hydrocephalus, risk factors, subarachnoid hemorrhage, traumatic brain injury

## Abstract

Traumatic brain injury (TBI) is associated with high morbidity and mortality rates despite all recent advances in its diagnosis and management. One of the main complications of TBI patients is post-traumatic hydrocephalus (PTH). Hydrocephalus after brain injury is considered to be multi-factorial, the exact underlying pathophysiologic mechanisms remain unclear, and any risk factors related to its development have remained ill-defined. In our current narrative review, we overview the proposed pathophysiologic mechanisms for PTH, PTH following decompressive craniectomy, de novo post-cranioplasty PTH, and review the pertinent literature regarding any PTH predisposing factors. Advanced patient’s age in adults and the presence of any comorbidities, the severity of the initial brain injury, as well as the presence of subarachnoid and/or intraventricular hemorrhages, the necessity for prolonged mechanical ventilation and the need for vasopressors’ administration have all been identified as predisposing factors for PTH. Furthermore, the necessity for decompressive craniectomy (DC), its extent and its proximity to the anatomical midline, the presence of subdural hygroma and/or interhemispheric hygroma following DC, as well as the presence of transcalvarial herniation seem to play a role in post-DC PTH. Moreover, there is an equipoise regarding the association of restorative cranioplasty (CP) and PTH. Several studies report development of PTH after CP with varying rates, while others postulate that CP may improve a pre-existing PTH. The role of timing of CP in the development or the improvement of pre-existing PTH remains highly controversial. Thorough knowledge and proper identification of predisposing factors may lead to early recognition and optimal treatment of PTH, thereby improving the overall functional outcome of TBI patients. On the contrary, misdiagnosis or delayed diagnosis of PTH may lead to severe disability and/or death.

## 1 Introduction

Traumatic brain injury (TBI) is a severe clinical-pathological condition. It represents a major global public health concern due to its associated high rates of mortality, morbidity, and long-term disability (1). It constitutes one of the most common causes of neurological impairment worldwide and is associated with a substantial compromise in quality of life among survivors (2, 3). Although advances in acute neurocritical care have improved survival, many patients, particularly those suffering moderate to severe TBI, develop secondary complications, which may affect functional recovery and long-term outcomes (4).

Post-traumatic hydrocephalus (PTH) is a well-recognized but incompletely understood complication of TBI (5). It is characterized by progressive accumulation of cerebrospinal fluid (CSF) and ventricular dilatation secondary to disruption of CSF circulation and absorption following severe head trauma (3, 6). This condition directly affects brain metabolism and function, and may lead to stagnation of neurological recovery, clinical deterioration, and poorer outcomes if not properly identified and treated promptly (7). Importantly, PTH is considered a potentially treatable complication of TBI, and its early detection and proper management can substantially improve overall patient prognosis (3, 6, 8).

The reported incidence of PTH varies widely in the literature, ranging from 0.7% to as high as 51.4% (7, 8). This wide variability is largely attributed to differences in diagnostic establishment, timing of diagnosis, imaging interpretation, and variations in nomenclature and classification systems (7, 9–11). Moreover, several studies have demonstrated a higher incidence of PTH among patients undergoing decompressive craniectomy (DC), suggesting that surgical decompression may play a role in its development (12, 13). In patients with moderate to severe TBI, ventriculomegaly is commonly observed during follow-up. For example, previous reports have shown that up to 31.6% of patients develop post-traumatic ventriculomegaly, with a smaller subset progressing to symptomatic PTH requiring ventriculoperitoneal (VP) shunt placement (5). However, despite extensive investigation, clear and consistent risk factors for the development of PTH have not been conclusively identified (14).

The pathophysiology of PTH is believed to be multifactorial and remains poorly understood (3). Proposed mechanisms include impaired CSF absorption due to subarachnoid hemorrhage, inflammation, fibrosis of arachnoid granulations, altered brain parenchyma compliance, and changes in cerebral blood flow following trauma and any associated surgical interventions (3). Distinguishing symptomatic PTH from post-traumatic ventriculomegaly secondary to cerebral cortical atrophy remains a major clinical challenge, particularly in patients with severe TBI (Glasgow Coma Scale <8). This diagnostic uncertainty contributes to the ongoing controversy regarding the surgical management of PTH after head injury (2).

Despite these challenges, satisfactory outcomes have been reported with CSF shunting in appropriately selected patients, emphasizing the importance of accurate diagnosis and timely treatment. Indeed, PTH is regarded as one of the leading causes of disability during rehabilitation following TBI and its proper management has a significant impact on functional recovery and overall outcome (15).

Although numerous studies have reported the incidence of PTH, relatively few have systematically evaluated potential risk factors that may identify patients at higher risk of developing PTH among TBI patients. A better understanding of such risk factors could improve surveillance strategies, facilitate early diagnosis, and optimize therapeutic decision-making and overall management. The purpose of our current study is to review and identify any risk factors associated with the development of PTH.

## 2 Materials and methods

Our current study is a narrative review of the literature focusing on risk factors associated with the development of post-traumatic hydrocephalus following TBI. Relevant studies were identified through searches of electronic databases, including EMBASE, PubMed and Scopus from January 1950 up to March 2026, using combinations of keywords such as “post-traumatic hydrocephalus,” “posttraumatic hydrocephalus,” “traumatic brain injury,” “head injury,” “decompressive craniectomy,” “cranioplasty,” “risk factors,” “predictors,” and “associated factors,” using Boolean operators where appropriate. Additional relevant publications were also identified by reviewing the reference lists of selected articles. Only articles published in English were included. Studies were considered eligible if they investigated the incidence, pathophysiology, or risk factors of PTH following TBI. Observational studies, cohort studies, case–control studies, systematic reviews, and meta-analyses were included. Case reports, small case series, conference abstracts, editorials, and studies focusing primarily on non-traumatic forms of hydrocephalus were excluded. Priority was given to studies with clear clinical relevance, including observational studies, systematic reviews, and meta-analyses, in order to provide a broad yet meaningful overview of the topic.

Given the variability in definitions, diagnostic criteria, and study designs across the literature, the findings were approached with a critical perspective (Table 1). Greater emphasis was placed on studies with larger patient cohorts, prospective methodology, longer follow-up periods, and clearly defined diagnostic criteria for PTH. Particular attention was also paid to important methodological limitations, including retrospective study design, selection bias, variability in diagnostic definitions and imaging criteria, differences in follow-up duration, and inadequate adjustment for potential confounding factors. Areas of ongoing debate, particularly the relationship between decompressive craniectomy, cranioplasty, and the development of PTH, were examined in the context of differences in study design, patient populations, and outcome measures. Rather than treating all studies equally, attention was paid to differences in methodology, patient populations, and reported outcomes. Whenever possible, findings from higher-level evidence were considered alongside observational data, while recognizing the limitations inherent to each type of study.

**Table 1:** Definitions of terms and conditions associated with CSF abnormalities following head injuries.

Term	Definition
Post-traumatic hydrocephalus (PTH)	Abnormal gradually progressive enlargement of the cerebral ventricles following traumatic brain injury, resulting from impaired cerebrospinal fluid (CSF) circulation and/or absorption (5).
Post-traumatic ventriculomegaly	Enlargement of the cerebral ventricles following TBI that is not necessarily associated with abnormal CSF dynamics. It is mainly associated with post-traumatic cortical atrophy (2)
Symptomatic PTH	A subset of PTH characterized by ventricular enlargement accompanied by clinical signs or symptoms attributable to hydrocephalus (10).
Subdural hygroma	CSF effusion in the subdural space, typically occurring after TBI or decompressive craniectomy due to disruption of arachnoid membranes and CSF circulation (48).
De novo post-cranioplasty hydrocephalus	New-onset of hydrocephalus following cranioplasty in patients who had no post decompressive craniectomy hydrocephalus (59).

Overall, the goal of this review is not simply to summarize the existing literature but to provide a balanced and clinically meaningful interpretation of the available evidence, highlighting areas of agreement, ongoing debate, and aspects that require further investigation.

## 3 PTH pathophysiologic mechanisms

The exact pathophysiologic nature of PTH remains ill-defined despite many theories proposed to explain its development. There is consensus that PTH has a multifactorial origin, and a growing body of evidence supports the concept that many of the proposed theories may synergistically explain the sequence of events leading to PTH.

It has been postulated that the presence of blood and its degradation products in the ventricular and/or the subarachnoid space may cause impaired CSF circulation and absorption (16, 17). This may be caused by direct mechanical obstruction of the normal CSF pathways. Moreover, the induction of an inflammatory process and activation of multiple inflammatory cascades may eventually lead to gliosis of the arachnoid granulations and the subarachnoid space (17). There is a growing body of evidence supporting the concept that TBIs induce inflammatory responses, which lead to fibrosis (17). Indeed, TBIs cause upregulation of transforming growth factor beta (TGF-*β*), a key mediator of fibrosis that modulates glial fibrillary acidic protein and a series of connective tissue growth factors (17). Similarly, previous studies have shown upregulation of neutrophil extracellular traps (NETs), which are another major contributor to fibrosis (17). Both these mechanisms amplify neuroinflammatory cascades and perpetuate impaired CSF absorption (17).

Moreover, the induced inflammatory processes may decrease lymphatic and glymphatic CSF drainage from the brain (16, 17). Animal studies have demonstrated that CSF drains into cervical and spinal lymphatic systems via pathways associated with cranial and spinal nerves (16, 17). The key role of the lymphatic CSF drainage along the olfactory nerve has been well established (17). Disruption of these lymphatic pathways elevates intracranial pressure and impairs CSF absorption (17). Previous studies have shown that a glymphatic system facilitates CSF movement from periarterial spaces and meningeal lymphatic vessels to the deep cervical lymph nodes (17). Aquaporin-4 (AQP-4), a water channel protein, plays a key role in the cerebral glymphatic system by facilitating exchange between CSF and the interstitial space, thereby providing waste clearance (mainly amyloid-*β* and Tau proteins) and neuroprotection (18). Experimental models have found that TBIs cause changes in AQP-4 levels, resulting in diffuse distribution and mispolarization (19). These changes impair the CSF glymphatic drainage, which further perpetuates the inflammatory response leading to brain edema formation, and to a vicious cycle of impaired CSF circulation and absorption (17).

It has been demonstrated that TBIs result in disruption of the blood–brain barrier (BBB), blood-CSF barrier, and brain-CSF barrier. Impairment of the BBB leads to ependymal tight junction breakdown, which promotes osmotic protein leakage into the CSF, water retention, and subsequent ventricular dilatation and potentially hydrocephalus (17). Likewise, disruption of the blood-CSF barrier induces activation and upregulation of matrix metalloproteinases and nuclear factor kappa-*β*, which weaken the ependymal tight junctions and facilitate leucocyte infiltration. This perpetuates the regional inflammatory response, induces further inflammatory cascades, resulting in chronic gliosis and impaired CSF circulation (17). Moreover, impairment of the brain-CSF barrier, caused by direct mechanical stress and/or inflammation of the ependymal cells, may promote vulnerability of the periventricular white matter. This zone hosts progenitor neural stem cells, which play a critical role in restorative neurogenesis and white matter repair. An insult to these cells depletes their restorative capacity, rendering the periventricular white matter chronically fibrotic and eventually gliotic, leading to the development and progression of hydrocephalus (17).

It has been postulated that cerebral pulsatility plays a key role in the development of hydrocephalus. It is well known that TBIs and traumatic SAH (tSAH) reduce cerebral compliance and increase arterial pulse pressure. This results in decreased vascular buffering mechanisms, further intensifying cerebral capillary pulsatility and disrupting normal CSF dynamics. These changes cause periventricular tissue damage and, along with disruption of the blood-CSF and brain-CSF barriers, induce depletion of progenitor neural stem cells in the periventricular white matter and eventually precipitate periventricular white matter gliosis and hydrocephalus (17, 20).

Moreover, recent evidence suggests that alterations in brain tissue properties may also contribute to PTH. Injury-related inflammation and structural damage can increase brain stiffness, making it more prone to deformation and permanent ventricular enlargement, even without markedly increased intracranial pressure (20). This supports the idea that hydrocephalus may partly reflect impaired brain integrity, in addition to disrupted CSF dynamics.

## 4 PTH predisposing factors

### 4.1 Patient’s factors

Several associated factors in patients have been implicated in the development of PTH. Numerous previously published studies have identified advanced age in adults, the presence of comorbidities, and persistent electrolyte abnormalities as PTH predisposing factors (21, 22). Medical comorbidities contributing to PTH development include arterial hypertension, diabetes mellitus, and any pre-existing neurological disorders, due to cerebral microvascular pathology, which is an independent risk factor for secondary CSF disturbance (23, 24). The presence of any electrolytic imbalances has been shown to affect CSF absorption via the arachnoid granulations (25). However, a clear association between persistent electrolytic changes, which are quite common among TBI patients, and the development of PTH has not been clearly established (26).

Advanced patient in adults age has consistently been identified as a predisposing PTH factor in previous publications (21, 27, 28). The reported increased PTH incidence may be associated with age-related pre-existing brain atrophy, reduced CSF absorption associated with degenerative venous changes, and decreased brain parenchymal compliance (21, 23, 29). It has to be also taken into consideration that elderly patients have poorer overall outcomes, while the TBI-associated mortality rate is very high (30, 31). This growing body of evidence supports the postulation that advanced age constitutes a predisposing factor for PTH among adult patients.

In the pediatric population, roughly 1% of children who experience TBIs will develop PTH, with the condition being more prevalent among children under 1 year of age (32). It has to be pointed out that the incidence of PTH among children undergoing DC is higher compared to adults. A systematic review and meta-analysis conducted in 2018 found that the incidence of PTH following DC in children is approximately three times higher than in adults (33).

### 4.2 Injury factors

Many injury-related factors have been associated with increased incidence of PTH. The severity of injury, as reflected by the admitting Glasgow Coma Scale (GCS) score, as well as other parameters such as prolonged loss of consciousness lasting more than 30 days, postoperative coma, and persistent pupillary abnormalities, has been reported to be associated with PTH development (10, 29, 34).

**Figure 1:**
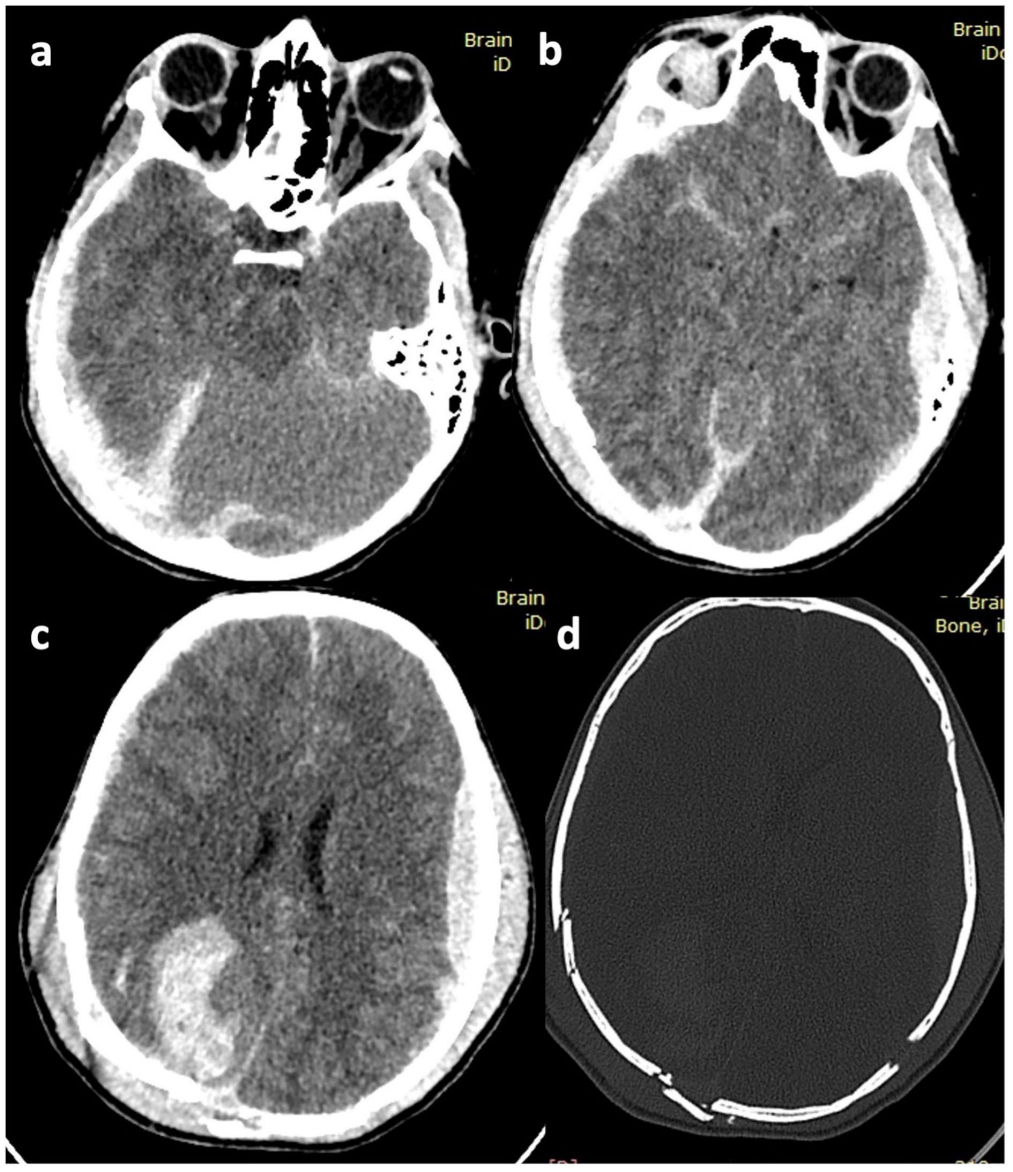
Head CT scan of a patient with severe traumatic brain injury showing: **(a)** diffuse brain edema, complete obliteration of the basal cisterns along with subarachnoid hemorrhage, **(b)** left acute subdural hematoma, **(c)** large right parietal intracerebral hematoma and adjacent cerebral contusions along with contralateral acute subdural hematoma, **(d)** multiple fractures of the cranial vault crossing the superior sagittal sinus on both sides.

The relationship between the admission GCS score and PTH has been widely studied, but the results remain conflicting. Several clinical investigators suggest that a lower GCS score at admission is associated with a higher risk of developing PTH (6, 35–37). These studies indicate that patients with more severe TBIs, as reflected by lower admission GCS scores, often present with complex intracranial pathologies that induce a strong inflammatory response and eventually ependymal and subarachnoid space gliosis, impair normal CSF circulation and absorption, and thus increase the likelihood of PTH development. The presence of epidural hematoma (EDH), a large SDH (maximal thickness >18 mm), tSAH, intracerebral hemorrhage (ICH), and intraventricular hemorrhage (IVH) has been associated with PTH development in previous studies (Figure 1) (29, 38, 39). Romualdo et al. found that SAH in the basal cisterns and cerebral contusions are independent risk factors for post-DC PTH (39). The presence of IVH, and both the thickness and distribution of tSAH, have been associated with the development of PTH, and when both are present, the likelihood of PTH is even higher (11). A recent systematic review and meta-analysis identified IVH as an independent, strong risk factor for PTH (37). Similarly, the presence of IVH has been identified as an independent predictor for post-DC PTH (29).

However, other previously published studies have found no significant association between admitting GCS score and PTH incidence, suggesting that GCS score may not be an independent risk factor (8, 27, 40). One possible explanation is that some severely injured patients with very low admitting GCS scores die before hydrocephalus develops, potentially diluting the apparent effect of poor GCS score on the PTH development risk. These findings indicate that, while the GCS score reflects overall injury severity, it may not consistently predict PTH across all patient populations. Overall, the pertinent literature demonstrates an equipoise regarding the effect of low GCS score upon admission and subsequent PTH development. Our clinical experience supports the concept that lower admitting GCS score is associated with the development of PTH.

### 4.3 Management factors

Surgical or other therapeutic interventions, management of side effects, or in-hospital systemic complications have been associated with PTH. These include respiratory, cardiac, urinary, and thromboembolic complications, septicemia, other systemic hematoma or hemorrhage, as well as other than PTH neurological complications (22, 34). Furthermore, the need for prolonged mechanical ventilation and elevated concentrations of inflammatory and molecular markers, such as NOD-like receptor family pyrin domain containing 3 (NLRP3), Matrix Metalloproteinase-9 (MMP-9), and Interferon-*γ* (IFN-γ), have been identified as predisposing factors for PTH (41).

**Figure 2:**
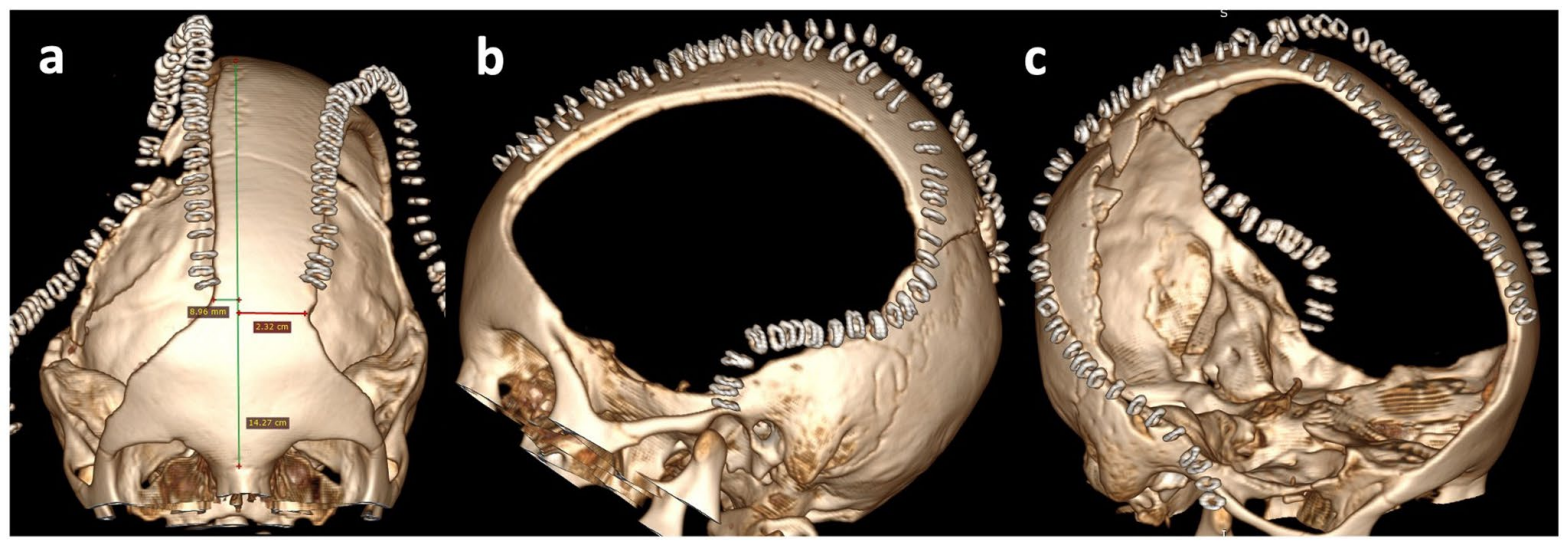
3-D reconstruction of postoperative head CT scan of a patient who underwent bilateral decompressive craniectomies for severe traumatic brain injury: **(a)** right-sided craniectomy’s medial edge is located very close to the anatomical midline (< 1 cm), while left-sided craniectomy is >2 cm from the midline, **(b)** left-sided craniectomy, **(c)** right-sided craniectomy.

Among the surgical interventions, DC constitutes the most important PTH predisposing factor (Figure 2) (2, 37, 42). Several clinical series have reported that approximately 60% of TBI patients undergoing DC required shunt surgery (8, 37, 42). In a large cohort of severe TBI patients the authors found that adults undergoing a DC was the only significant predictor for VP shunting (43). DC has been associated with impaired CSF absorption resulting from compromised venous drainage and increased cerebral pulsatility due to decreased cerebral compliance (7). The removal of the bone flap alters normal intracranial biomechanics, allowing transmission of the pressure pulse through the open cranium, leading to flattening of the physiological dicrotic intracranial pressure waveform (44, 45). This reduction in pulsatile force decreases CSF outflow toward the arachnoid granulations, and impairs cerebral blood flow, CSF hydrodynamics, and CSF glymphatic clearance (3, 6, 38, 45–47).

**Figure 3:**
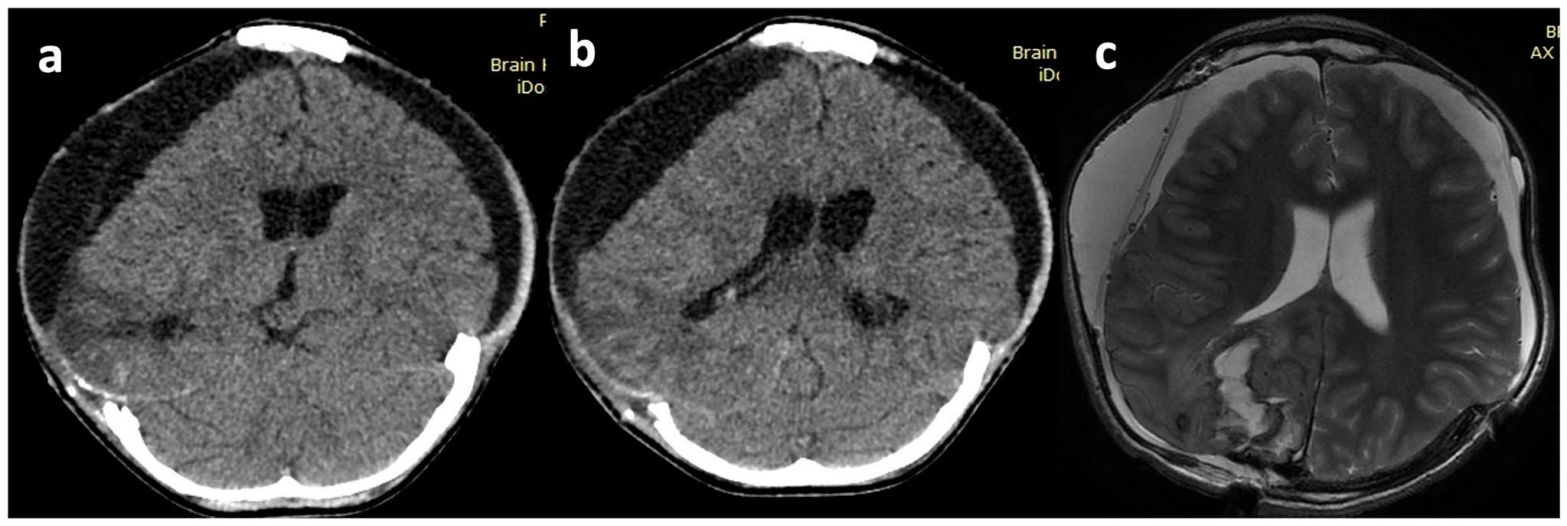
Traumatic bilateral subdural hygromas on: **(a,b)** head CT scan and **(c)** T2 weighted image brain MRI of a patient undergoing decompressive craniectomy for severe traumatic brain injury, 1 month ago.

The size of the performed DC seems to play a role in the development of PTH, with larger size or bilateral DCs predisposing to a higher incidence of PTH (7, 8, 12, 37, 42). It has also been reported that the proximity of the DC’s medial edge (≤ 25 mm) to the anatomical midline predisposes to PTH (12, 37). A possible pathophysiologic mechanism is mechanical compression of the cortical bridging veins draining into the superior sagittal sinus, which may compromise venous outflow and CSF absorption, thereby promoting CSF accumulation and PTH development. Furthermore, the presence of convexity subdural and/or interhemispheric hygromas has been identified as a predictor for post-DC PTH (Figure 3) (12, 13, 36, 37, 39). Hygromas typically form within approximately 1 week after surgery and peak at 3–4 weeks postoperatively (48). Their presence indicates disrupted CSF circulation and impaired absorption, which, along with decreased brain edema, decreased compliance, and increased arterial pulsatility, may lead to PTH (49–51). Therefore, the presence of any hygromas should raise the suspicion of PTH development, especially among minimally responsive patients (13, 48, 49). Likewise, transcalvarial brain herniation (TCH) is another condition associated with post-DC PTH (13, 39, 52). It has been demonstrated that both a higher TCH volume and the presence of subdural hygromas are associated with an increased risk of post-DC PTH (53). Even the time interval between TBI and DC performance seems to play a role in the development of post-DC PTH (39). It has been reported that early performed DC is associated with a higher incidence of post-DC PTH (39).

It should be noted, however, that some studies suggest that DC itself may not be an independent risk factor for PTH (23, 48). Several clinical investigators have postulated that while hydrocephalus can occur following DC, its development is linked to the severity of the initial brain injury and other confounding factors, including SAH and IVH, and the presence of subdural hygromas (36, 54). It has been postulated that patients requiring VP shunting were also more likely to experience unfavorable neurological outcomes, suggesting that it is the primary injury and its complications rather than DC that cause PTH (35). Our clinical experience is consistent with the literature supporting the association between DC and PTH. The question is whether post-DC PTH has underlying pathophysiological mechanisms different from those of PTH. Further experimental research and more robust clinical data may answer this question in the future.

**Figure 4:**
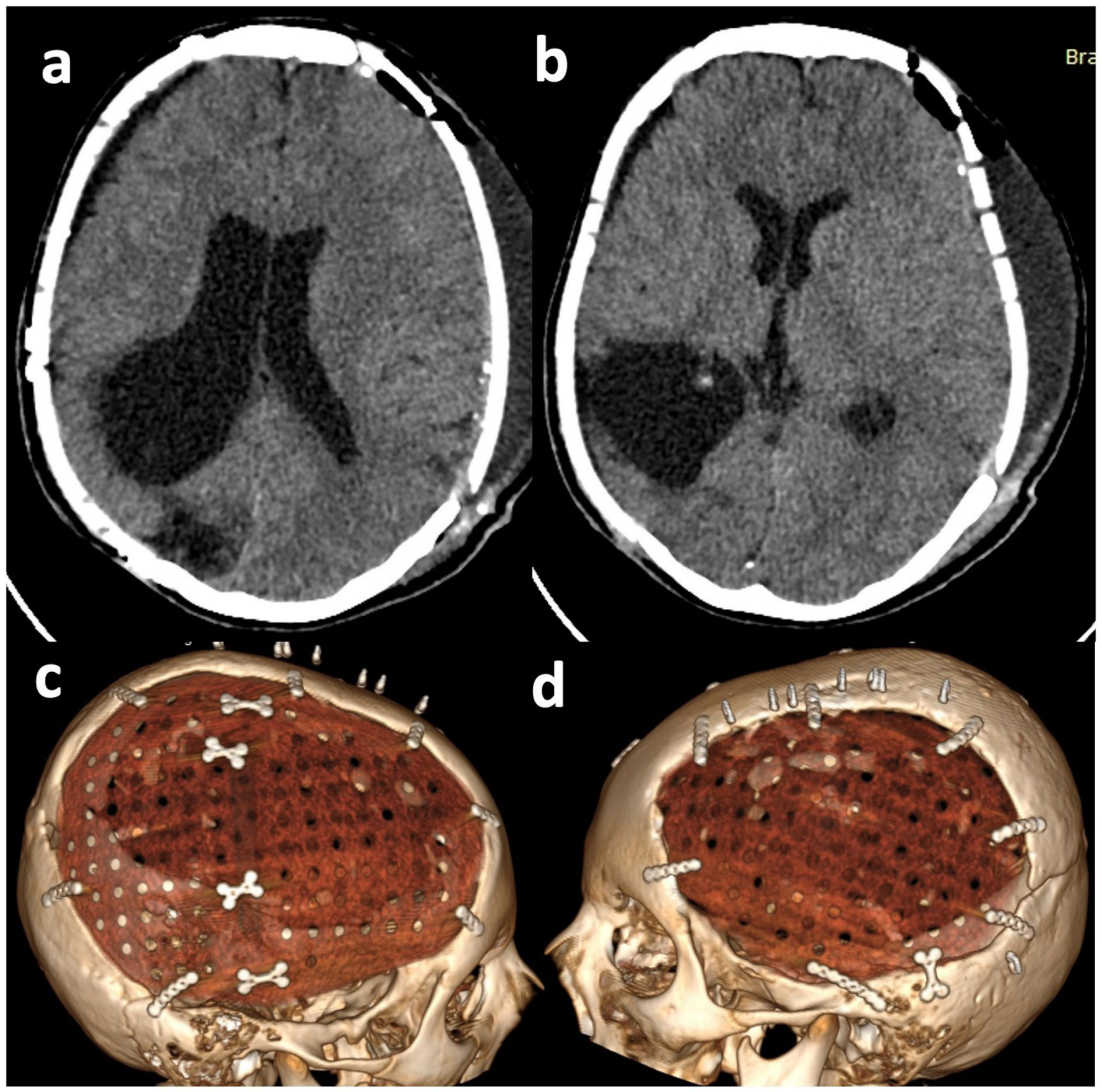
Head CT images **(a,b)** of a patient with severe traumatic brain injury, who underwent restorative cranioplasty following decompressive craniectomy. 3-D reconstruction of postoperative head CT scan **(c,d)** of the same patient showing right-side **(c)** and left-side **(d)** restoration.

Another controversial issue is the relationship of post-DC PTH, *de novo* PTH, and cranioplasty (CP). *De novo* PTH has been associated with the performance of a restorative CP, with varied reported rates, based on the study population and the timing of CP (Figure 4) (53, 55–59). According to the German Cranial Reconstruction Registry, newly diagnosed post-CP PTH occurred in 2% of the cohort (60), whereas the corresponding percentage reported in a systematic review and meta-analysis was 8.1% (61). In another clinical series, *de novo* post-CP PTH was observed in 2.8% of patients, whereas post-DC PTH present before CP likely reflected the sequelae of the primary injury and DC performance rather than CP performance (50). The timing of restorative CP has been investigated as a potential factor influencing post-DC PTH incidence, but evidence remains inconsistent. Several retrospective studies and systematic reviews reported an increased risk of post-DC PTH in patients undergoing early CP (within 90 days from DC), suggesting that early intervention may alter CSF dynamics and increase the possibility of PTH and the need for permanent CSF diversion (53, 55, 56, 62–65). However, other studies suggest no significant difference between early and delayed CP (21, 48, 66).

On the other hand, other investigators have postulated that CP may improve preexisting, post-DC PTH. A previously published clinical study reported that half of the patients with symptomatic post-DC PTH experienced complete symptom resolution after CP (67). These contradictory findings in the literature may reflect differences in study populations, mixed cases of ventriculomegaly and PTH, or an undetected post-DC PTH prior to CP. Our clinical experience supports the idea that restorative CP may improve post-DC PTH in many cases. However, the impact of CP timing on the incidence of *de novo* PTH, as well as on the evolution of post-DC PTH, remains to be defined.

## 5 Conclusion

Post-traumatic hydrocephalus constitutes a common and potentially serious complication following severe TBI. Differentiating it from post-traumatic ventriculomegaly and recognizing its development are critical, particularly among patients with identified high-risk factors, such as advanced age, low admitting GCS score, presence of tSAH, IVH, or undergoing DC. In these patients when a DC is inevitable, avoidance of bilateral DCs and approximation of the anatomical midline (≤ 25 mm) are advocated, while high vigilance is required when subdural hygromas and/or transcalvarial herniations are present. A thorough understanding of these risk factors enables clinicians to maintain heightened awareness, ensuring focused monitoring and timely appropriate surgical intervention. Close follow-up during the rehabilitation phase is essential, as PTH may emerge or become clinically apparent during this period, and optimal management can improve functional outcome and overall recovery.
